# *Bryocyclopsasetus* sp. n. and the presence of *Bryocyclopsmuscicola* (Menzel, 1926) from Thailand (Crustacea, Copepoda, Cyclopoida, Cyclopidae)

**DOI:** 10.3897/zookeys.793.25005

**Published:** 2018-10-29

**Authors:** Santi Watiroyram

**Affiliations:** 1 Division of Biology, Faculty of Science, Nakhon Phanom University, Nakhon Phanom 48000, Thailand Nakhon Phanom University Nakhon Phanom Thailand

**Keywords:** Caves, copepods, freshwater, redescription of *Bryocyclopsmuscicola*, new species, taxonomy

## Abstract

The description of *Bryocyclopsasetus***sp. n.** and the record of *B.muscicola* (Menzel, 1926) from Thailand are presented. The new species is most similar to *B.maewaensis* Watiroyram, Brancelj & Sanoamuang, 2012, the cave-dwelling species described from northern and western Thailand. They share morphological characteristics, such as the free margin of the anal operculum which is ovated and serrate, the same setae and the spines formulae on P1–P4Exp-2 (setae: 5.5.5.4; spines: 3.3.3.3) and Enp-2 of P1–P2, P4 (setae formula 3.4.3) in both sexes. The new species is easily distinguished from *B.maewaensis* due to typical divergent caudal rami, the absence of coxal seta on P1, and the absence of blunt-tipped setae on P2–P3Exp-2. A dichotomous key to the species of *Bryocyclops* group I *sensu*Lindberg (1953) is proposed.

## Introduction

**Figure 1.**
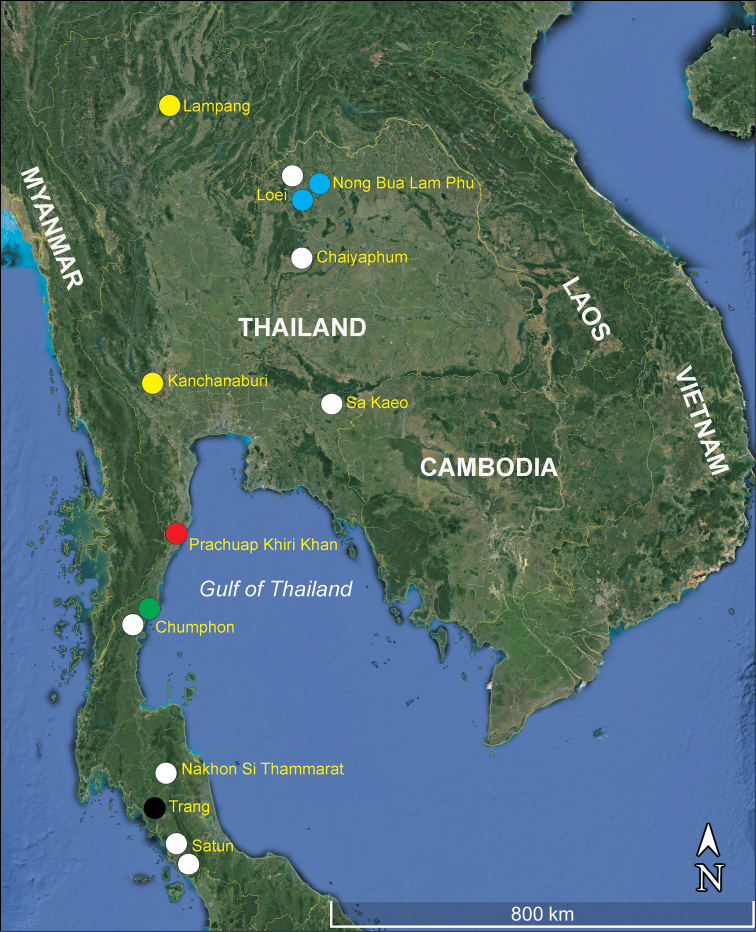
The geographical distribution of the genus *Bryocyclops* Kiefer, 1927 in Thailand. The coloured circles (O) indicate approximate location of species: yellow, *B.maewaensis* from Lampang and Kanchanaburi Provinces; white, *B.muscicola* from Loei, Chaiyaphum, Sa Kaeo, Chumphon, Nakhon Si Thammarat, and Satun Provinces; blue, *B.maholarnensis* from Loei and Nong Bua Lam Phu Provinces; red, *B.asetus* sp. n. from Prachuap Khiri Khan Province; green, *B.muscicoloides* from Chumphon Province; black, *B.trangensis* from Trang Province.

The genus *Bryocyclops* Kiefer, 1927 is the most abundant genus of Cyclopidae Rafinesque, 1815 from Thailand and it is widely distributed in Southeast Asia. At present, six species have been reported from Thailand: *B.maewaensis* Watiroyram, Brancelj & Sanoamuang, 2012; *B.maholarnensis* Watiroyram, Brancelj & Sanoamuang, 2015; *B.muscicoloides* Watiroyram, 2018; *B.trangensis* Watiroyram, 2018; *B.muscicola* (Menzel, 1926) and *B.asetus* sp. n. (Figure 1). All species have so far been found in caves and are presumed to be endemic to Thailand, except *B.muscicola*, which frequently has been found in both subterranean and non-subterranean habitats (Jocque et al. 2013; present study). *Bryocyclopsasetus* sp. n. belongs to group I *sensu*Lindberg (1953) because it shares the armature of the first to the fourth swimming legs. Group I is the most diverse group of the genus, consisting of *B.anninae* (Menzel, 1926) from Guam, Hawaii, Vanuatu, and Indonesia (Java, Sumatra); *B.ankaratranus* Kiefer, 1955 and *B.mandrakanus* Kiefer, 1955 from Madagascar; *B.apertus* Kiefer, 1935, *B.difficilis* Kiefer, 1935, and *B.elachistus* Kiefer, 1935 from Kenya; *B.chappuisi* Kiefer, 1928 from Indonesia (Java); *B.phyllopus* Kiefer, 1935 from Congo, Ethiopia, Kenya; and *B.maewaensis* and *B.asetus* sp. n. from Thailand (Dussart and Defaye 2006; Watiroyram et al. 2012). *Bryocyclopsabsalomi* Por, 1981 from Israel which has not been assigned into a species group by Por (1981) and Dussart and Defaye (2006), could be part of another species of group I (except the P1 coxa lacking inner seta). However, most group I members, even other *Bryocyclops* members, have been inadequately described by authors who usually focus on the anal operculum and selected legs. Indeed, the fine details (i.e. microcharacters) on the body ornamentation (cephalothorax scar, posterior margins, pores or grooves) and the structure of swimming legs (transformed setae and spines, and spiniform processes, especially the fourth leg and the male’s third leg) are often considered to be important characters in the most recently described species. These characteristics are useful for determining closely related species and degrees of leg modifications (Reid 1999; Fiers and van Damme 2017; Watiroyram 2018). The new species, *B.asetus* and the new record of *B.muscicola* from Thailand are presented and their morphological and physical adaptations briefly discussed. An updated key to species of *Bryocyclops* group I *sensu*Lindberg (1953) is also provided.

## Materials and methods

Water samples were collected using plankton and hand nets (60 µm) and preserved in 70% ethanol. Adult animals were sorted and dissected under an Olympus SZ51 stereomicroscope in a mixture of glycerol and 70% ethanol (ratio ~ 1:10 v/v) and pure glycerol. Dissected specimens were mounted in pure glycerol and sealed with nail polish. All appendages and body ornamentation were examined under an Olympus compound microscope (CX31) at 1000× magnification. Drawings were made with a drawing tube (an Olympus U-Da) mounted on a compound microscope. The final versions of the drawings were made using the CORELDRAW^®^ 12.0 graphic program. Specimens for scanning electron microscopy (SEM) were dehydrated in an ethanol series (70%, 80%, 90%, 95%, 100%, and 100% absolute ethanol) for 15 min each concentration. Specimens were dried in a critical point dryer using liquid carbon dioxide as exchange medium. Dried specimens were mounted on stubs using adhesive tape under stereomicroscope. Specimens were coated with gold in a sputter-coater. The SEM photographs were made using a scanning electron microscope (LEO 1450 VP).

The following abbreviations are used throughout the text and figures:

**A** aesthetasc;

**Enp** endopod;

**Exp** exopod;

**Exp/Enp-n** exopodal segment n/endopodal segment n;

**P1–P6** swimming legs 1–6;

**S** seta/setae;

**Sp** spine/spines

The appendage terminology follows Huys and Boxshall (1991). Specimens were deposited at the Natural History Museum, London, United Kingdom (**NHMUK**) and at the Nakhon Phanom University, Faculty of Science, Thailand (**NPU**).

## Taxonomic section

### Order Cyclopoida Rafinesque, 1815

#### Family Cyclopidae Rafinesque, 1815

##### Genus *Bryocyclops* Kiefer, 1927

###### Bryocyclops asetus sp. n.

Taxon classificationAnimaliaCyclopoidaCyclopidae

http://zoobank.org/FAA0B3EA-9B61-44E1-8836-309C3C7524BD

**Figure 2.**
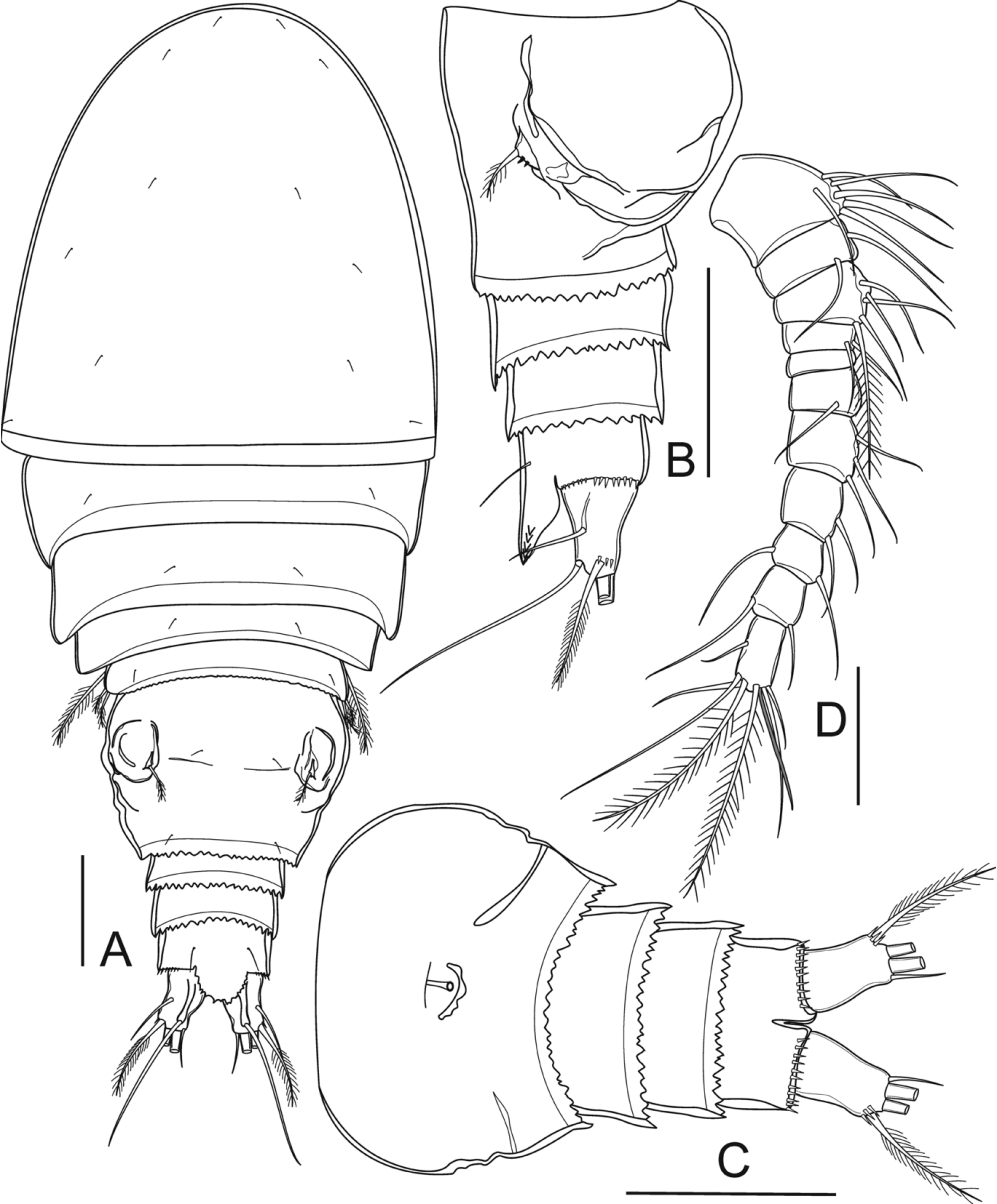
*Bryocyclopsasetus* sp. n., female: **A** habitus, dorsal view **B** urosome (without pediger V), lateral view **C** urosome (without pediger V), ventral view **D** antennule. Scale bar: 50 µm.

**Figure 3.**
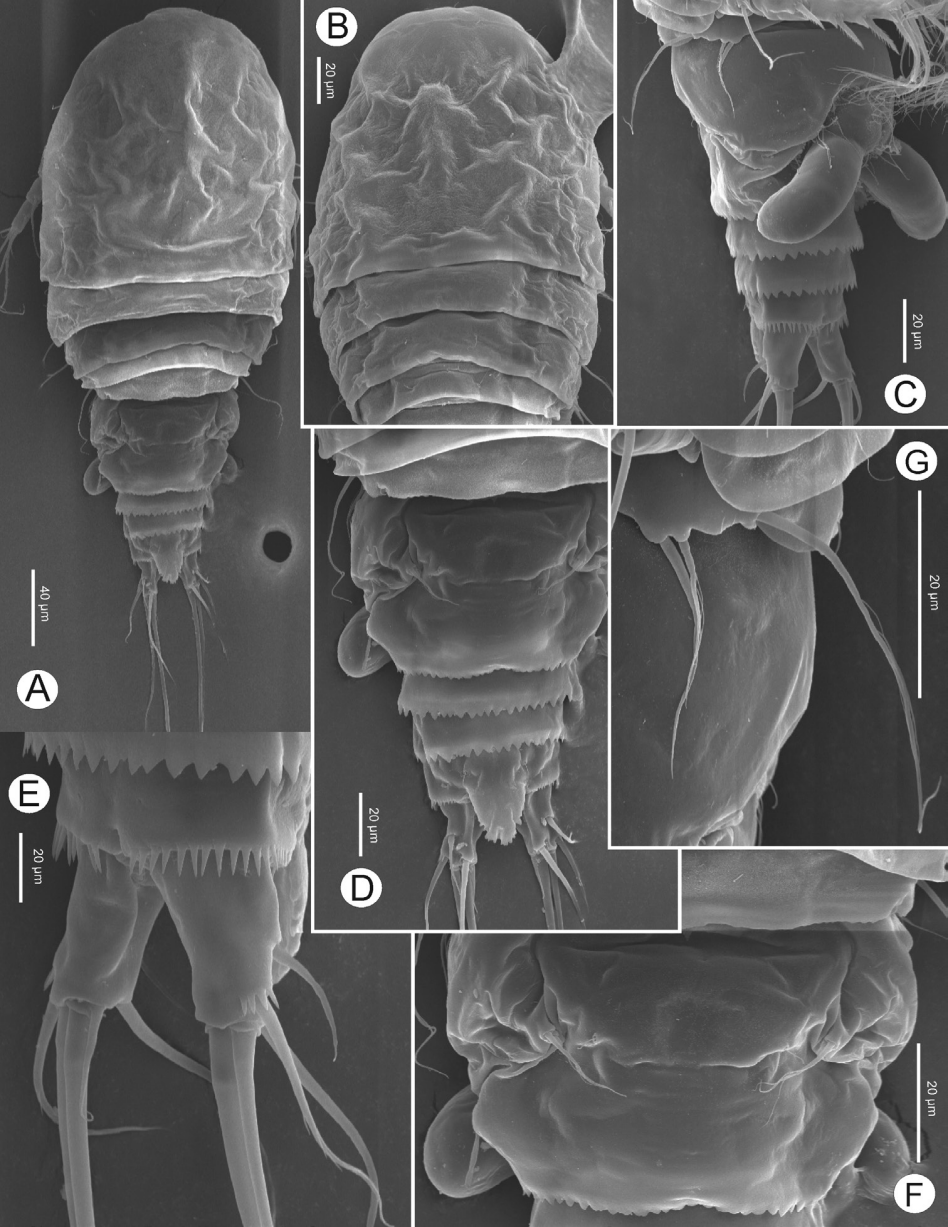
*Bryocyclopsasetus* sp. n., SEM photographs of female: **A** habitus, dorsal view **B** prosome, dorsal view **C** urosome (genital double-somite with spermatophore), ventrolateral view **D** urosome, dorsal view **E** caudal ramus, ventrolateral view **F** genital double-somite, dorsal view **G**P5.

**Figure 4.**
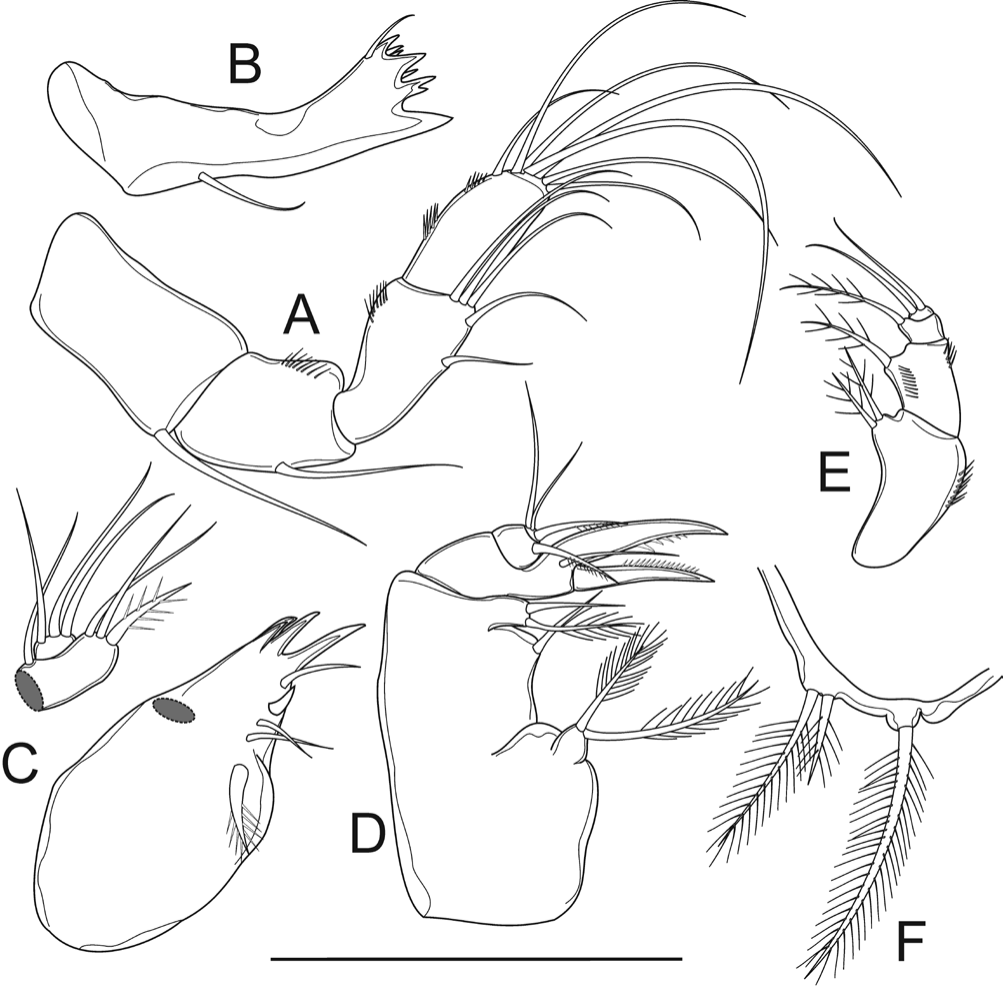
*Bryocyclopsasetus* sp. n., female: **A** antenna **B** mandible **C** maxillule **D** maxilla **E** maxilliped **F**P5. Scale bar: 50 μm.

**Figure 5.**
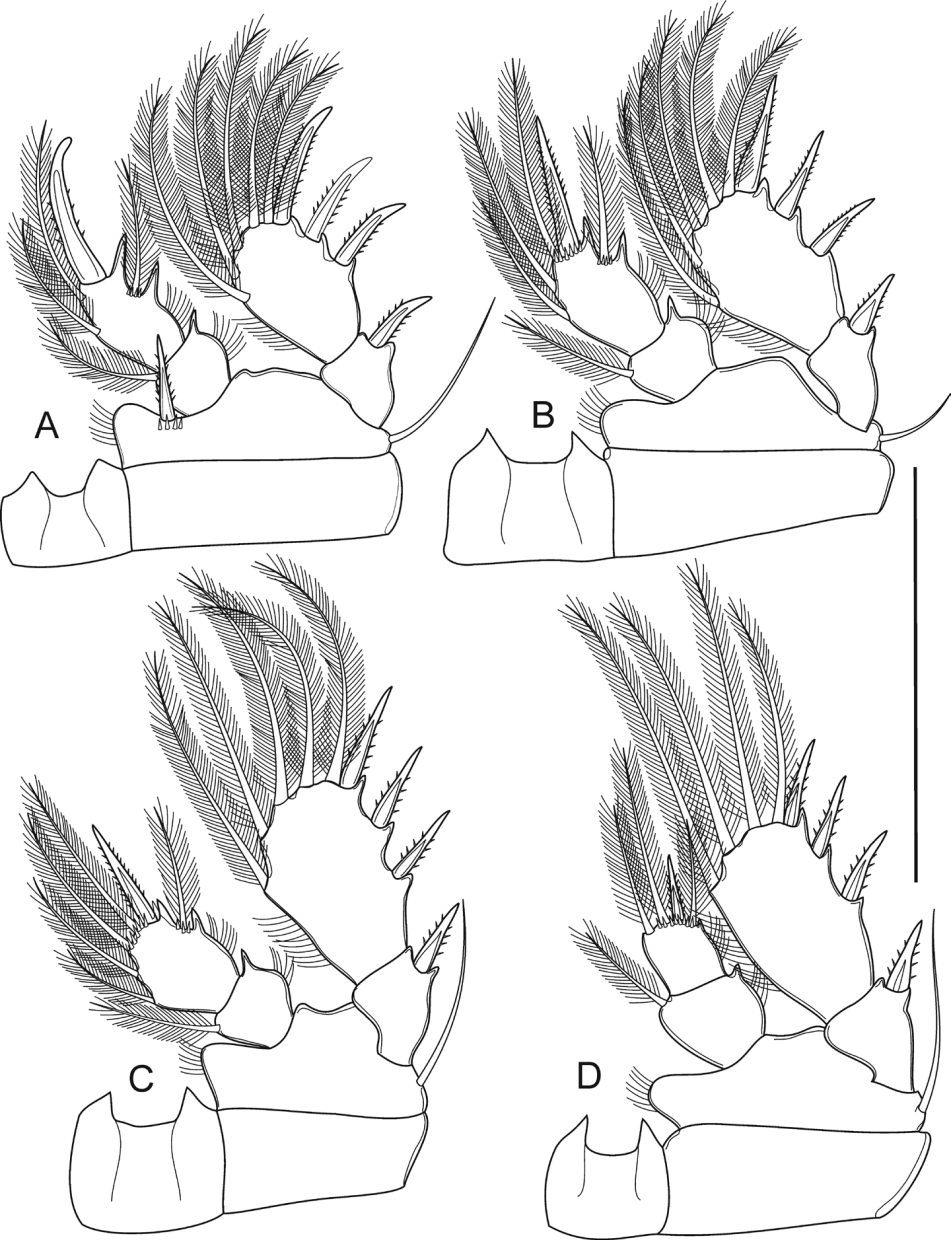
*Bryocyclopsasetus* sp. n., female: **A**P1**B**P2**C**P3**D**P4. Scale bar: 50 μm.

**Figure 6.**
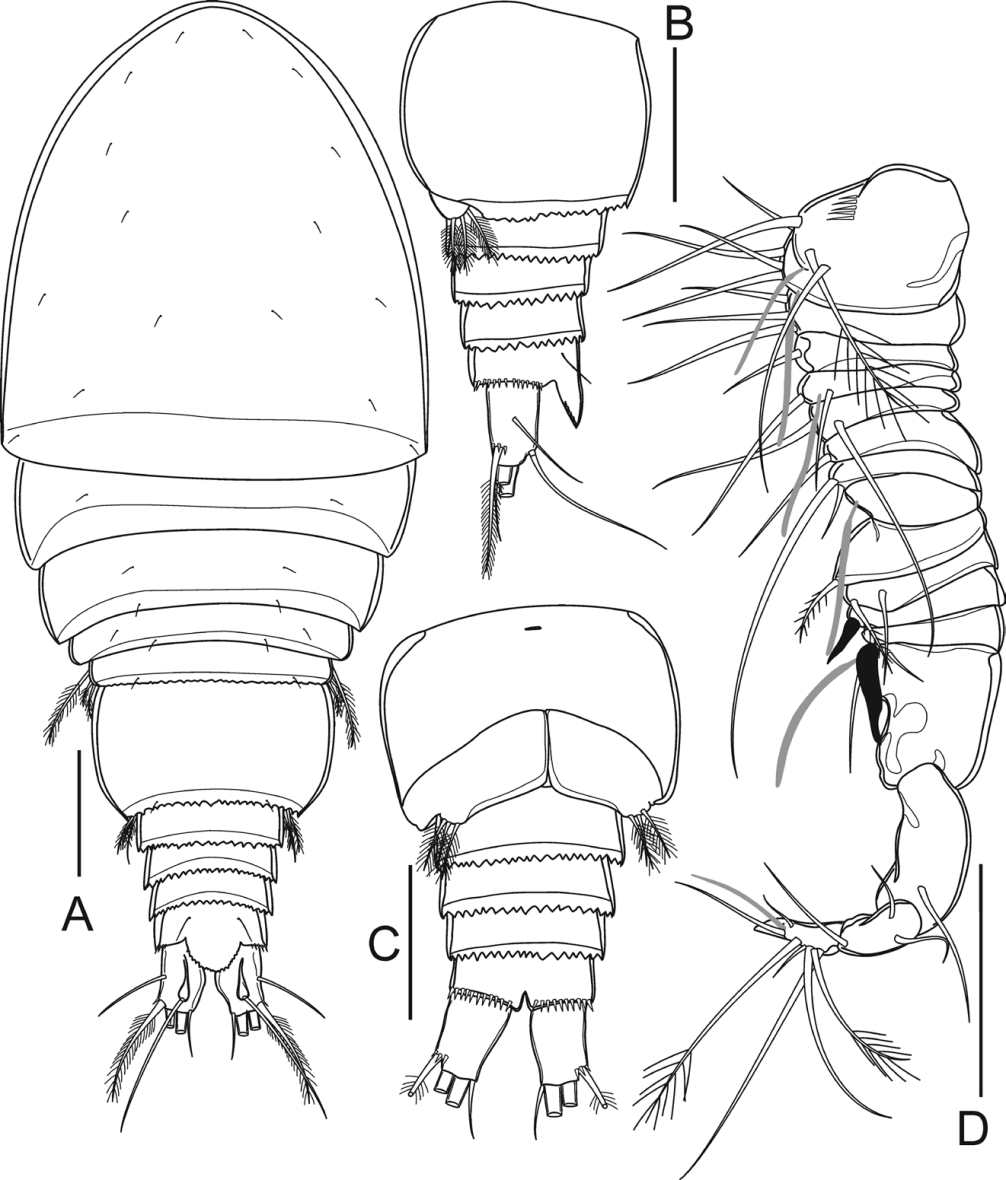
*Bryocyclopsasetus* sp. n., male: **A** habitus, dorsal view **B** urosome (without pediger V), lateral view **C** urosome (without pediger V), ventral view **D** antennule. Scale bar: 50 µm.

**Figure 7.**
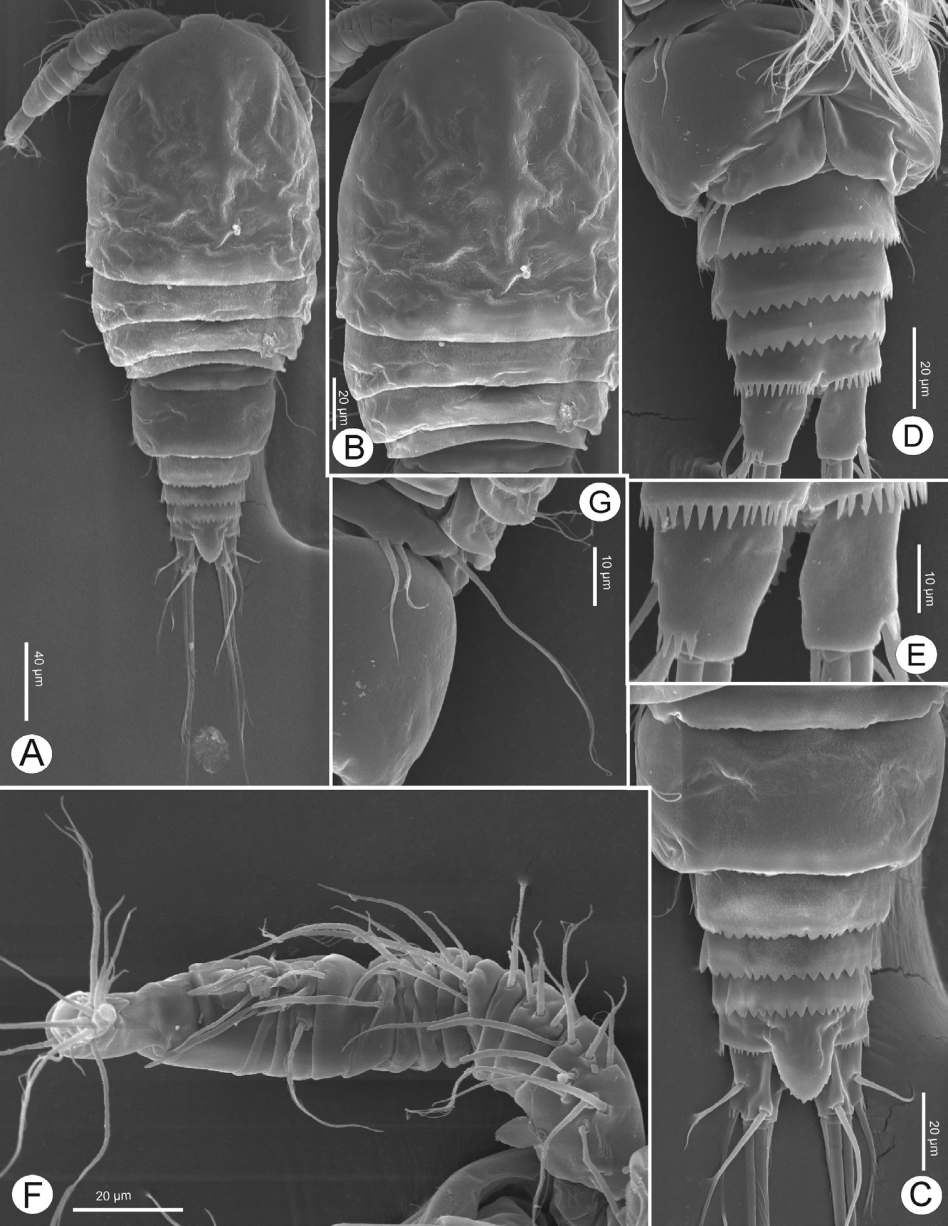
*Bryocyclopsasetus* sp. n., SEM photographs of male: **A** habitus, dorsal view **B** prosome, dorsal view **C** urosome, dorsal view **D** urosome, ventral view **E** caudal ramus, ventral view **F** antennule.

**Figure 8.**
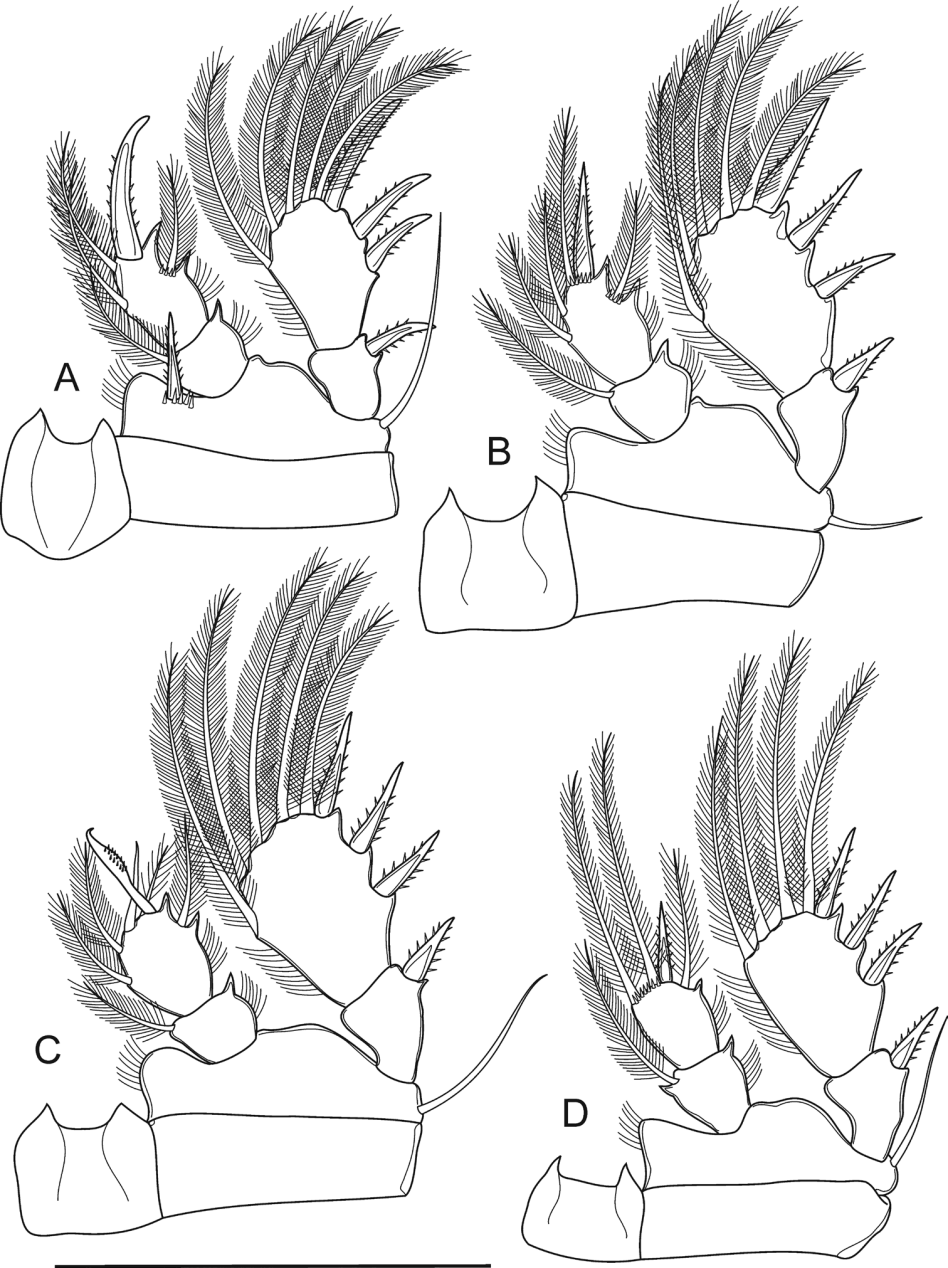
*Bryocyclopsasetus* sp. n., male **A**P1**B**P2**C**P3**D**P4. Scale bar: 50 μm.

Figs 2 , 3 , 4 , 5 , 6 , 7 , 8

####### Type locality.

A rimstone pool in Sai Cave, Khao Daeng Subdistrict, Kui Buri District, Prachuap Khiri Khan Province, western Thailand; 12°10'46"N; 100°00'26"E, altitude: 107 m above sea level.

####### Material examined.

Holotype: one adult male, NHMUK 2018.1043, dissected and mounted on one slide; allotype: one adult female, NHMUK 2018.1044, dissected and mounted in one slide; paratypes: one ovigerous female, two adult females and three adult males, NHMUK 2018.1045-1050, preserved in 70% ethanol in 1.5 ml microtube and three adult females and three adult males, NPU 2018–003, preserved in 70% ethanol in 1.5 ml microtube; all specimens collected on 1 December 2016 by author.

####### Differential diagnosis.

Anal operculum ovate and serrate. P1–P4 with acute projections on distal margin of intercoxal sclerite; no inner coxal seta. Basis of P1 with inner spine. P1–P4 with two-segmented Exp and Enp. Setal and spine formula of P1–P4Exp-2 as follows: 5.5.5.4; 3.3.3.3; with no blunt-tipped setae; P1–P4Enp-2 as 3.4.5.3 and 1.1.1.1. Male P3Enp-2 with six elements, including one transformed spine and seta: transformed spine with acute tip; slightly swollen in medians part, armed with strong spinules; transformed seta bare, strong.

####### Description of adult female.

Preserved specimens colourless. Nauplius eye and refractile points on integument absent. Body length (Figs 2A, 3A) (n = 5) measured from anterior margin of rostrum to posterior margin of caudal rami 398–415 μm (mean 406 μm, n = 5); greatest width at distal part of cephalothorax 156–169 μm (mean 164 μm, n = 5); body length/width ratio 2.5. Cephalothorax completely fused to pediger I, without dorsal scar. Prosome length 245–248 μm (mean 246 μm, n = 5), urosome length 165–169 μm (mean 167 μm, n = 5); prosome/urosome length ratio 1.5. Prosomites with posterior dorsal margins smooth; urosomites serrated; pediger V finely serrated, and genital double-somite and two later urosomites coarsely serrated, both dorsally and ventrally (Fig. 3B, D). Genital double-somite (Figs 2A–C, 3F) symmetrical; length 73–76 μm (mean 75 μm, n = 5), width 98–100 μm (mean 99 μm, n = 5); 1.3 times wider than broad. Pair of sclerotized structures dorsolaterally, remnant of ancestral segment both dorsally and ventrolaterally; copulatory pore situated midventrally. Anal somite (Figs 2A–C, 3D–E) with strong spinules along entire posterior margin; pair of long sensilla above base of anal operculum. Anal operculum ovate, coarsely serrated, extending over three-fourths of caudal ramus length.

Caudal rami (Figs 2A–C, 3D–E) divergent, slightly tapering on distal half, 1.5 times as long as wide. Each ramus with six setae (seta II–VII): anterolateral (II) seta bare, slightly shorter than ramus; posterolateral (III) seta pinnate, 1.3 times as long as ramus, with strong spinules near venterolateral insertion; outer terminal (IV) and inner terminal (V) setae longest, pinnate, with fracture plane; terminal accessory (VI) seta shortest, bare, 0.5 times as long as ramus; dorsal (VII) seta located on distal margin of dorsal keel, bare, 2.2 times as long as ramus, with articulate insertion.

Antennule (Figure 2D) 11-segmented do not reach posterior margin of cephalothorax. Armature formula: 1(6S), 2(2S), 3(5S), 4(2S), 5(0), 6(1S), 7(3S), 8(2S), 9(2S), 10(2S), 11(7S+1A).

Antenna (Figure 4A) uniramous, consisting of coxobasis, and three-segmented Enp. Coxobasis with one smooth seta on distal inner corner. Enp-1–3 with 1, 5, and 7 setae respectively; with row of spinules on outer margin.

Mandible (Figure 4B) with six strongly chitinized teeth; dorsal seta on gnathobase. Palp reduced to single seta.

Maxillule (Figure 4C) with four strongly chitinized teeth on precoxal arthrite; inner margin with four bare setae and one plumose seta. Palp two-segmented: proximal segment with distal plumose seta; two bare setae distally; one bare seta dorsally; distal segment with three bare setae.

Maxilla (Figure 4D) with precoxal endite with two pinnate setae. Coxa with two endites: proximal endite with one bare seta; distal endite with one pinnate, and one bare setae. Basis with two strong claw-like expansions, bare seta close to its base. Two-segmented Enp, Enp-1 with unipinnate seta; Enp-2 with one unipinnate seta, two bare setae.

Maxilliped (Figure 4E) syncoxal endite with two strong spiniform setae, and row of spinules on anterior surface. Basis with spiniform seta, two rows of spinules on anterior surface. Two-segmented Enp; Enp-1 with one pinnate seta; Enp-2 with two bare setae.

P1–P4 (Fig. 5A–D) biramous, with two-segmented Exp and Enp. Intercoxal sclerites with no ornamentation, with acute projections on distal margin. P1 with group of tiny spinules at insertion of inner spine. All setae on P1–P4Exp-2 with normal tips. P1–P4Enp-1 with spiniform process on outer distal corner. Outer seta and inner proximal seta on P1Enp-2 as long as spine, shorter than inner distal seta. Outer seta and inner proximal seta on P2Enp-2 as long as spine, shorter than two inner distal setae. Proximal inner seta on P3Enp-2 as long as spine, shorter than the rest of segment; outer seta slightly longer than spine, followed by two inner medial setae and apical seta. Apical seta on P4Enp-2 longest, 2.5 times as long as spine, followed by inner and outer seta. Spine and setal formula of P1–P4 as follows (seta in Arabic numerals, spine in Roman numerals for outer-inner or outer-apical-inner seta/spine):

**Table:** 

	Coxa	Basis	Exp		Enp	
			1	2	1	2
P1	0-0	1-I	I-0	III-2-3	0-1	1-I-2
P2	0-0	1-0	I-0	III-2-3	0-1	1-I+1-2
P3	0-0	1-0	I-0	III-2-3	0-1	1-I+1-3
P4	0-0	1-0	I-0	III-2-2	0-1	1-I+1-1

P5 (Figs 3G, 4F) fused to pediger, represented by three pinnate setae on small prominence: dorsal seta longer than two ventral setae; outer seta longer than inner one.

P6 (Figs 2A–B, 3F) situated laterodorsally next to heavily sclerotized structure on genital double-somite: with one short pinnate seta dorsally; two tiny spinules next to it ventrally element on small plate.

Adult females with pair of egg sacs (Figure 12A), each with two eggs, with a mean diameter of 64 μm (n = 8).

####### Description of adult male.

Body length (Figs 6A, 7A) measured from anterior margin of rostrum to posterior margin of caudal rami 369–373 μm (mean 371 μm, n = 5); smaller than female. General segmentation and ornamentation (Figs 6A–C, 7A–C) similar to female, with five-segmented urosome. Anal operculum (Figure 7C) more finely serrated than in female. Antennae, mouthparts, P1–P2, P3Exp-2, and P5 (Figs 7G, 8A–C) similar to those of female, except P2Enp-2 with longer inner medial seta.

Antennule (Figs 6D, 7F) 16-segmented, geniculate. Armature formula as follows: 1(7S+2A), 2(3S), 3(1S), 4(2S+1A), 5(2S), 6(1S), 7(2S), 8(1A), 9(1S), 10(2S), 11(1Sp), 12(0), 13(2S+1A+1Sp), 14(1S), 15(3S), 16(7S+1A).

P3 (Figure 8C) intercoxal sclerite with acute projections on distal margin. Enp-1 with inner pinnate seta. Enp-2 with outer pinnate seta; transformed apical spine with hook-like tip, thin pinnate seta; inner strong bare seta, two inner pinnate setae. Transformed apical spine with several spinules at 2/3 length of it; less-produced medial portion; with short, acute tip.

P4 (Figure 8D) intercoxal sclerite with acute projections on distal margin. Coxa without inner seta. Basis and Exp-1 with outer seta and spine, respectively. Exp-2 with three outer spines and two pinnate apical and inner setae. Enp-1 with inner pinnate seta and spiniform process on inner and outer distal margins. Enp-2 with apical spine and three long pinnate setae: apical and inner setae equal in length, 3.0 times as long as spine; outer seta 1.5 times longer than spine.

P6 (Figs 6B, C, 7D) reduced to simple plate, represented by three subequal pinnate setae.

####### Variability.

Two out of five examined females have round rather than acute distal margins on intercoxal sclerite of P1.

####### Etymology.

The specific name *asetus* refers to the one of the primary characteristic that discriminates the new species from other species of group I *sensu*Lindberg (1953) by the first swimming leg without an inner coxal seta.

####### Geographical distribution.

This species is currently known only from Sai Cave, Khao Daeng Subdistrict, Kui Buri District, Prachuap Khiri Khan Province, Thailand.

####### Remarks.

The new species is most similar to *B.maewaensis*, found in caves in northern and western Thailand. They share the same morphological characteristics, such as the free margin of anal operculum ovate and serrate, the same setae and spines formula on P1–P4Exp-2 (setae: 5.5.5.4; spines: 3.3.3.3) and the same setae formula on P1–P4Enp-2 (3.4.5.3) in both sexes, except the male P3Enp-2 of *B.asetus* sp. n. which has five instead of four setae. The new species is clearly differentiated from *B.maewaensis* by its typical divergent caudal rami, the absence of a coxal seta on P1 and the absence of blunt-tipped setae on P2–P3Exp-2. Female P4Enp-1 of *B.asetus* sp. n. is larger than Enp-2 and has no spiniform process on the inner distal margin, which is present in *B.maewaensis*. Female P4Enp-2 of the new species has three long slender setae which are longer than the apical spine on the same segment, but *B.maewaensis* has robust and short setae that are approximately as long as spine. Male P3Enp-2 of the new species has a different transformed spine compared to *B.maewaensis*: the expansion is less expressed and is armed with strong spinules in the new species, but *B.maewaensis* has a well-expressed expansion and no ornamented surface.

###### Bryocyclops muscicola

Taxon classificationAnimaliaCyclopoidaCyclopidae

(Menzel, 1926)

Figs 9 , 10 , 11

####### Material examined.

One dissected adult female and male mounted separately on one slide from each (seven) caves: Pha Pu Cave (NPU 2018–004-005), Na-Or Subdistrict, Muang District, Loei Province, 17°33'21"N; 101°43'39"E, altitude: 247 m, collected on 18 August 2014; Chang Phueak Cave (NPU 2018–006-007), Banna Subdistrict, Muang District, Chumphon Province, 10°26'46"N; 99°02'06"E, altitude: 104 m, collected on 10 August 2015; Mae Nang Songsi Cave (NPU 2018–008-009), Hin Tok Subdistrict, Ron Phibun District, Nakhon Si Thammarat Province, 08°14'45"N; 99°52'01"E, altitude: 45 m collected on 23 October 2015; Khao Tiphon Cave (NPU 2018–010-011), Thung Wa Subdistrict, Thung Wa District, Satun Province, 07°05'10"N; 99°47'53"E, altitude: 46 m collected on 24 October 2015; Khao Jean Cave (NPU 2018–012-013), Khlong Khut Subdistrict, Muang District, Satun Province, 06°38'32"N; 100°05'77"E, altitude: 38 m collected on 25 October 2015; Kaew Cave (NPU 2018–014-015), Laem Thong Subdistrict, Phakdi Chumphon District, Chaiyaphum Province, 15°58'27"N; 101°24'36"E, altitude: 394 m, collected on 19 September 2016; Khao Maka Cave (NPU 2018–016-017), Sala Lamduan Subdistrict, Muang District, Sa Kaeo Province, 13°47'10"N; 101°56'51"E, altitude: 121 m, collected on 5 November 2017. Four adult males and four adult females from Chang Phueak Cave mounted on one stub for SEM analysis (NPU 2018–018). All specimens collected by author.

####### Description of adult female.

**Figure 9.**
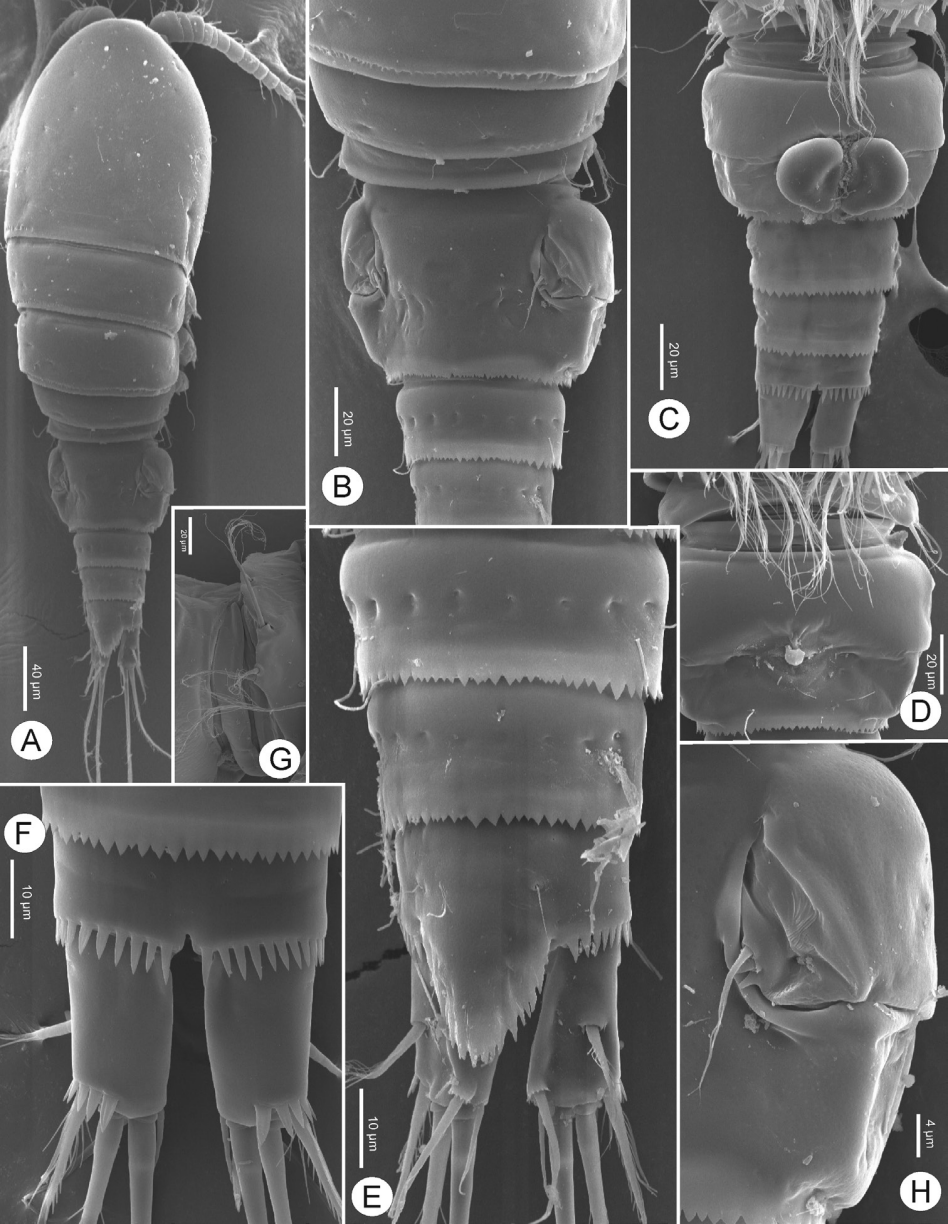
*Bryocyclopsmuscicola* (Menzel, 1926), SEM photographs of female: **A** habitus, dorsal view **B** pedigers III–V, genital double-somite, two later urosomites, dorsal view **C** urosome (genital double-somite with spermatophore), ventral view **D** genital double-somite, ventral view **E** urosome (without pediger V and genital double-somite), dorsal view **F** anal somite and caudal rami, ventral view **G**P5**H**P6.

Body length (n = 5) excluding caudal setae, 481–491 μm (mean 486 μm), width 154–156 μm (mean 155 μm), body length/width ratio approximately 3.0 (Figs 9A, 11A). Prosome length 290–293 μm (mean 292 μm), urosome length 208–213 μm (mean 210 μm); prosome/urosome length ratio 1.4. Body surface with refractile points from cephalothorax to genital double-somite. Cephalothorax with dorsally incorporated scar, pair of dorsolateral body pits. Prosomites with smooth posterior dorsal margins; pedigers I-IV with serrated lines above posterior margins; pedigers I and II with dorsolateral body pits. Urosomites with dorsally serrated posterior margins; finely serrated on pediger V; urosomites coarsely serrate both dorsally and ventrally (Fig. 9B, C, E). Genital double-somite (Fig. 9B–D) symmetrical, 1.6 times as long as wide. Pair of sclerotized structures dorsolaterally, single copulatory pore midventrally. Urosomite 3–4 (Figure 9E) with body pits on dorsolateral surface. Anal somite (Fig. 9E–F) with strong spinules along entire posterior margin; pair of long sensilla above base of anal operculum. Anal operculum ovate, coarsely serrated and reaches over three-fourths of caudal ramus.

Caudal rami (Fig. 9E–F) parallel. Ramus 1.6 times as long as wide; with well-developed dorsal keel. Each ramus with four (seta II–V) pinnate setae and two (seta VI–VII) bare setae: posterolateral (III), outer terminal (IV) and dorsal (VII) setae with spinules above its insertion; setae IV and V (inner terminal seta) with breaking plane.

P1–P4 (Fig. 11B–E) biramous with two-segmented Exp and Enp but P4 with one-segmented Enp. Intercoxal sclerites with acute projections on distal margin. P2–P3Exp-2 with two blunt-tipped setae (apical and inner seta) (Figure 11C). P1–P3Enp-1 with spiniform process on outer distal corner. P4Enp with spiniform process on mid-outer margin. Spine and setal formula of P1–P4 as follows (seta in Arabic numerals, spine in Roman numerals for outer-inner or outer-apical-inner seta/spine):

**Table:** 

	Coxa	Basis	Exp		Enp	
			1	2	1	2
P1	0-1	1-I	I-0	III-2-3	0-1	1-I-2
P2	0-0	1-0	I-0	III-2-3	0-1	1-I+1-1
P3	0-0	1-0	I-0	III-2-3	0-1	1-I+1-3
P4	0-0	1-0	I-0	III-2-2	1-I+1-2	-

P5 (Figure 9G) completely fused to pediger V, represented by three pinnate setae: dorsal seta longest, on small prominence; two ventral setae completely fused with pediger, inner seta shortest.

P6 (Figure 9H) located on genital double-somite at 1/2 length, dorsolaterally; represented by one short pinnate seta and two tiny spinules on small plate.

Adult females with pair of egg sacs (Figure 12B), each with two eggs, with a mean diameter of 50 μm (n = 8).

Spermatophore (Figure 9C) bean-shaped, paired, visible on genital double-somite mid-posteroventrally.

**Figure 10.**
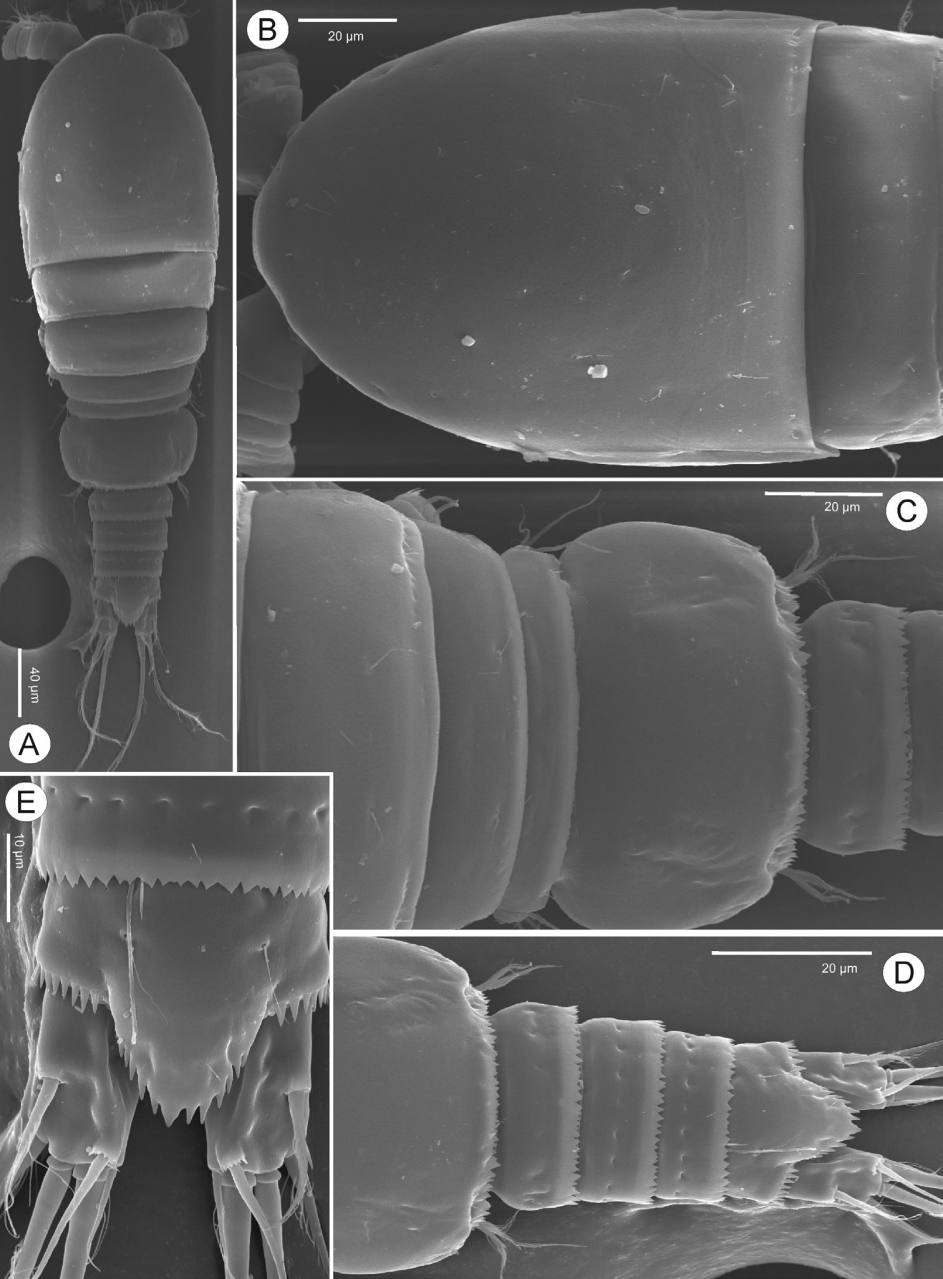
*Bryocyclopsmuscicola* (Menzel, 1926), SEM photographs of male in dorsal view **A** habitus **B** cephalothorax and pediger II **C** pedigers III–V, genital somite and later urosomites **D** genital somite and later urosomites **E** anal somite and caudal rami.

**Figure 11.**
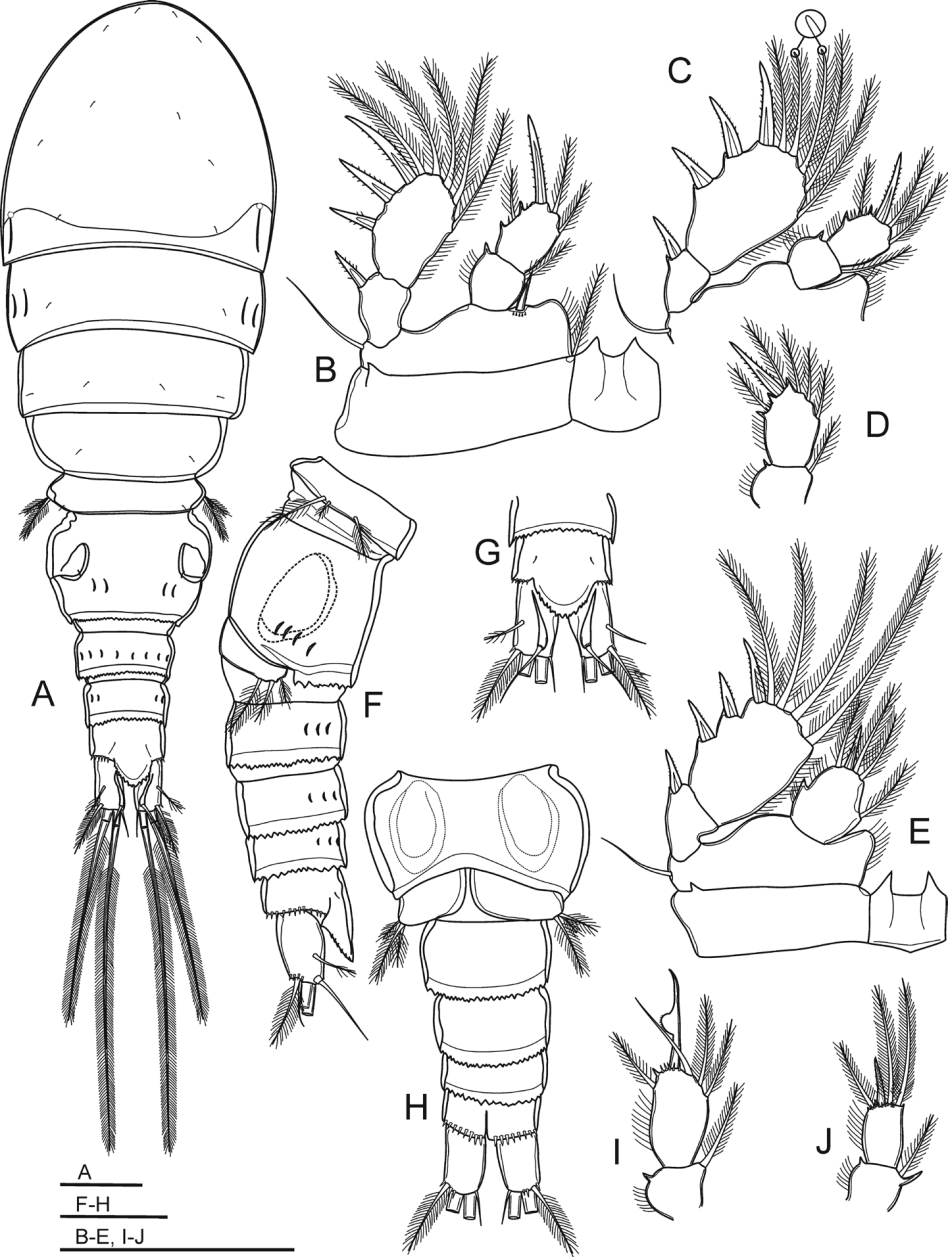
*Bryocyclopsmuscicola* (Menzel, 1926), female (**A–E**) and male (**F–J**) **A** habitus, dorsal view **B**P1**C**P2**D**P3Enp**E**P4**F** urosome, lateral view **G** anal somite and caudal rami, dorsal view **H** urosome (without pediger V), ventral view **I**P3Enp**J**P4Enp. Scale bar: 50 µm.

**Figure 12.**
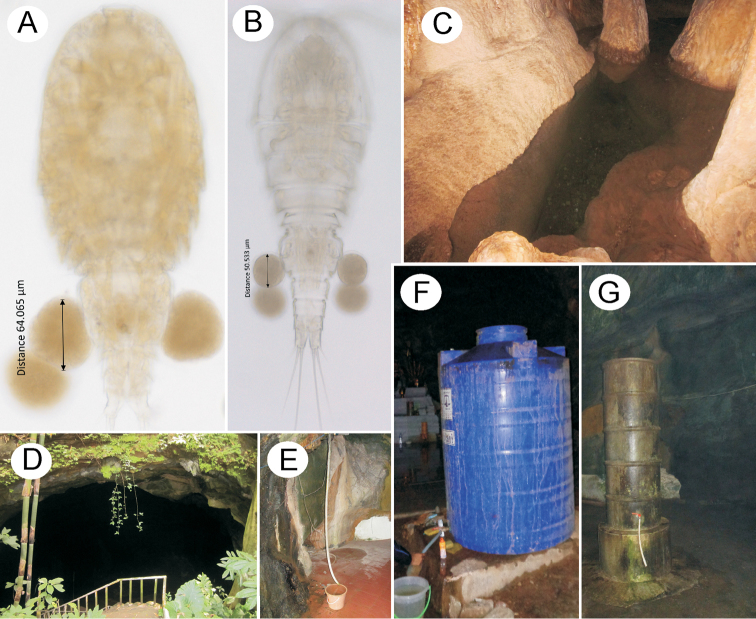
*Bryocyclopsasetus* sp. n. (**A, C**) and *B.muscicola* (Menzel, 1926) (**B, D–G**) **A–B** photographs in light microscopy showing eggs **C–G** microcavernicolous habitats from different zones **C** pool **D–E** water container collecting drip water from cliff (water container not shown in **D**) and stalactite **F–G** water tank (**C, F** in dark zone **G** in transition zone **D, E** in entrance zone).

####### Description of adult male.

Body length (Figs 10, 11F–H) smaller than female with total body lengths of 432–440 μm (mean 436 μm), prosome/urosome length ratio 1.3 (n = 5). Body ornamentation, armature of P1–P2, P3–P4Exp-2, and P5 similar to those of females. P3 (Figure 11I) with two-segmented Enp, Enp-1 with inner pinnate seta and spiniform process on outer distal margin. Enp-2 with three inner setae (distalmost seta bare, two others pinnate), transformed apical spine and outer pinnate seta. Transformed spine with medial-produced, semi-circular shape with spinules on swollen portion; with long distance between swollen portion and its tip; tip acute. P4 (Figure 11J) with two-segmented Enp; Enp-1 with inner pinnate seta and spiniform process on outer and inner distal margins; spiniform process strong and well-expressed on inner margin. Enp-2 with apical spine and three long pinnate setae, apical and inner seta subequal in length, 2.5 times as long as spine, outer seta shortest, 1.5 times longer than spine. P6 (Figure 11F, H) reduced to simple plate, represented by three unequal pinnate setae.

####### Geographical distribution.

*Bryocyclopsmuscicola* is the most widely distributed species within the genus *Bryocyclops*, which is mainly found in Southeast Asia with one record finding from North America (Florida, Reid 1999). To date, the species has been found in Indonesia (Java, Sumatra) and Thailand, including the north eastern region (Loei and Chaiyaphum provinces), the eastern region (Sa Kaeo Province), the southern region (Chumphon, Nakhon Si Thammarat, and Satun provinces) (Menzel 1926; present study, see Figure 1).

####### Remarks.

*Bryocyclopsmuscicola* belongs to *Bryocyclops* group II *sensu*Lindberg (1953) (Dussart and Defaye 2006). All specimens of *B.muscicola* collected from caves in Thailand are consistent in terms of the details of their legs, anal operculum, and body ornamentation as observed by Menzel (1926) and Reid (1999). Some differences are noted on caudal rami and prosome: (1) the caudal ramus of Thailand’s specimens have tiny spinules at the insertion of the dorsal seta (VII) instead of the lateral seta (III) in both sexes, and the males have an obviously longitudinal keel on their rami that is absent in the Florida’s specimens; (2) the cephalothorax and pedigers II-IV of Thailand’s specimens have serrated dorsal frills above their posterior margins, a frill only present on pedigers II-IV in the Florida specimens.

## Discussion

Most members of the genus *Bryocyclops* have been previously found in semi-subterranean rather than subterranean habitats, such as moist moss, wet soils, leaf litter, and phytotelmata (Reid 1999, 2001). Some known species are only described from representatives of the male or the female and not described enough precisely (Dussart and Defaye 2006). After examining specimens from different regions of Thailand, *Bryocyclops* are among the most common cave-dwelling cyclopoids living in the epikarst zone. Currently, there are six species from Thailand and one species from Israel (*B.absalomi* Por, 1981) (see introduction). All species are presumed to have a restricted distribution and are also probably endemic to the areas (counties) in which they were first described. In this study, *B.muscicola* was for the first time found in subterranean environment in water containers filled directly with dripping water from stalactites or cliffs at the transition and the entrance zones (Fig. 12D, E, G). They have also been found in abundance in water tanks located in the dark zone of some caves (Figure 12F). Therefore, *B.muscicola* can be considered a stygophilous species as it successfully lives in the subterranean in both natural and artificial habitats.

*Bryocyclopsasetus* sp. n. shares body ornamentation with members of group I (*B.anninae*; *B.maewaensis*; and *B.phyllopus*) and group II (*B.muscicola*; *B.caroli* Bjornberg, 1985; *B.campaneri* Rocha & Bjornberg, 1987; *B.muscicoloides* Watiroyram, 2018, and *B.trangensis* Watiroyram, 2018) according to the following characters: (1) the presence of dorsal serrated frills on the posterior margin of their prosomites (cephalothorax, pedigers II-IV of *B.asetus* sp. n., and *B.maewaensis*; pedigers II–IV of *B.campaneri*, *B.muscicola*, and *B.muscicoloides*; pediger III of *B.caroli*); (2) the presence of body groove on prosomites and urosomites (cephalothorax, pedigers II and IV, genital double-somite, and two subsequent urosomites of *B.asetus* sp. n., *B.campaneri*, *B.muscicola*, and *B.anninae*; cephalothorax, genital double-somite, and two subsequent urosomites of *B.muscicoloides*; genital double-somite of *B.trangensis*); and (3) the presence of cephalothorax scare (*B.asetus* sp. n., *B.maewaensis*, *B.phyllopus*, *B.muscicola*, *B.campaneri*, *B.muscicoloides*, and *B.trangensis*).

*Bryocyclopsabsalomi* Por, 1981 has sensory pits on cephalothorax, which were illustrated in SEM photographs (see Por 1981: fig. 23). This feature is observable under both scanning electron and light microscopes (i.e., show dark spots or light spots in SEM or light photographs, respectively). These pits were described with different terminology by earlier authors, and are here called refractile points, another characteristic of body ornamentation in the genus *Bryocyclops*. The SEM photograph in Figure 9H confirms the possession of refractile points on genital double-somite of *B.muscicola*, which usually have more coarse points on the cephalothorax and later pedigers. These cuticular points have been known in all previously described species from Thailand (*B.maewaensis*, *B.maholarnensis*, *B.muscicoloides*, *B.trangensis*) but are not present in *B.asetus* sp. n., and seem to be reported in all its congeners, or even in other cyclopoids and harpacticoids such as in *Elaphoidellabromeliaecola* (Chappuis, 1928), in caves from Thailand (see Reid 1999 for review of this characteristic; Watiroyram et al. 2012, 2015; Watiroyram 2018; pers. obs.).

Fiers and van Damme (2017) noted that the presence of spurs on the caudal surface near the inner distal margin of P4Enp in the genus *Bryocylops* indicated the degree of modified endopods. In this view, the new species and *B.anninae* probably have less unmodified P4Enp by the absence of spurs on Enp-1. Compared to the original figures of members in group I *sensu*Lindberg (1953), they also showed less modified P4Enp, except *B.maewaensis*, which has triangular spurs on the inner distal corner (see Watiroyram et al. 2012: figs 4D, 6D). Actually, only the females of group I have two-segmented P4Enp (showing less modified P4Enp) whereas other groups have more modified P4Enp by representing only single segment. The female P4Enp of other *Bryocyclops* species were modified or reduced into single segment, and the remnants of ancestral segments (i.e. outer spiniform processes on proximal segment) are represented by spurs or spiniform processes on outer margins such as *B.muscicola*, and *B.trangensis*. Two other Thai species, *B.muscicoloides* and *B.maholarensis*, show more reduced P4Enp by lacking processes on the outer margins. Generally, the female P4Enp of its congeners has one spine and four setae, resulting in the fusion of Enp-1 (one seta) and Enp-2 (one spine and three setae), whereas *B.maholarensis* has only two setae on the smaller segment. According to the above-mentioned morphology, *B.muscicoloides* and *B.maholarensis* are apparently most adapted to subterranean habitats on the fourth leg, compared to its congeners, by shortening the segment and reducing the setae on their legs (Suárez-Morales 2015).

*Bryocyclopsasetus* sp. n. has strongly divergent and robust caudal rami, which have never been observed in other members of the genus. *Bryocyclopsmaewaensis* has short and robust setae on the female P4Enp, almost as long as the spine on the same segment, compared to those in other *Bryocyclops* species, which are slender and never shorter than spine. Brancelj (2009) remarked that divergent and robust caudal rami, and short and strong seta on the caudal rami and their legs, are presumed to be important morphological adaptations for efficient movement in narrow spaces and drifting prevention in epikarstic species. Peculiar adaptations on caudal rami seem to have diversified *B.asetus* sp. n. from its epigean lineage. In contrast to *Parastenocaris* Kessler, 1913, caudal adaptation of vertical drift prevention is not a result of epikarstic stress but is presented as an epigean ancestor and tends to be a preadaptation for epikarstic species (Cottarelli et al. 2012). The presence of pseudosomite anterior to the genital double-somite in interstitial species was considered to provide enhanced movement in a narrow space by increasing the flexibility of the urosome (Huys and Boxshall 1991): *B.muscicola* and *B.correctus* Kiefer, 1960 (synonym of *Haplocyclopspauliani* Kiefer, 1955 in the sense of Fiers 2002) also have pseudosomites, probably a preadaptation to living in both epigean and hypogean habitats. A reduction in the number of eggs and egg size also results in physiological adaptation to trophic stress, which is common in stygobiotic species (Cottarelli et al. 2012): the new species shares stygobiotic specialization with *B.muscicola*, a stygophilic species which was observed to have a reduced number of eggs per ovigerous females. However, *B.asetus* sp. n. has a larger egg size, which is an indication of stygobiotic adaptation for subterranean habitats (Fig. 12A–B).

### Keys to species of group I *sensu*Lindberg (1953)

**Females**: *B.anninae* (Menzel, 1926); *B.chappuisi* Kiefer, 1928; *B.difficilis* Kiefer, 1935; *B.elachistus* Kiefer, 1935; *B.maewaensis* Watiroyram, Brancelj & Sanoamuang, 2012; *B.phyllopus* Kiefer; *B.asetus* sp. n.

**Table:** 

1	Anal operculum triangular, with acute tip	**2**
–	Anal operculum ovate or semi-circular, with round tip	**4**
2	Posterior margin of anal operculum serrate	*** B. chappuisi ***
–	Posterior margin of anal operculum smooth	**3**
3	Terminal accessory (VI) seta on caudal ramus short, not reaching beyond a fracture plane of inner terminal (V) seta	*** B. anninae ***
–	Terminal accessory (VI) seta on caudal ramus long, reaching well beyond a fracture plane of inner terminal (V) seta	*** B. phyllopus ***
4	Posterior margin of anal operculum finely serrate	*** B. elachistus ***
–	Posterior margin of anal operculum coarsely serrate	**5**
5	P1 coxa without seta on inner distal corner	***B.asetus* sp. n.**
–	P1 coxa with seta on inner distal corner	**6**
6	P4Enp-2 with three short and robust setae, as long as spine	*** B. maewaensis ***
–	P4Enp-2 with three long and slender setae, relatively longer than spine	*** B. difficilis ***

**Males**: *B.ankaratranus* Kiefer, 1955; *B.apertus* Kiefer, 1935; *B.difficilis* Kiefer, 1935; *B.elachistus* Kiefer, 1935; *B.maewaensis* Watiroyram, Brancelj & Sanoamuang, 2012; *B.mandrakanus* Kiefer, 1955; *B.phyllopus* Kiefer, 1935; *B.asetus* sp. n.

**Table:** 

1	Anal operculum triangular, with acute tip	*** B. phyllopus ***
–	Anal operculum ovate or semi-circular, with round tip	**2**
2	Posterior margin of anal operculum smooth	*** B. apertus ***
–	Posterior margin of anal operculum serrate	**3**
3	P1 coxa without seta on inner distal corner	***B.asetus* sp. n.**
–	P1 coxa with seta on inner distal corner	**4**
4	Posterior margin of anal operculum finely serrate	*** B. elachistus ***
–	Posterior margin of anal operculum coarsely serrate	**5**
5	Anal operculum less-produced, shorter than half of caudal ramus	*** B. difficilis ***
–	Anal operculum well-produced, longer than half of caudal ramus	**6**
6	P3Enp-2 with one spine and four setae	*** B. maewaensis ***
–	P3Enp-2 with one spine and five setae	**7**
7	The expansion of transformed spine of P3Enp-2 serrate	*** B. ankaratranus ***
–	The expansion of transformed spine of P3Enp-2 smooth	*** B. mandrakanus ***

## Supplementary Material

XML Treatment for Bryocyclops asetus

XML Treatment for Bryocyclops muscicola
